# Change in Hepatitis A Seroprevalence among U.S. Children and Adolescents: Results from the National Health and Nutrition Examination Survey 2003–2006 and 2007–2010

**DOI:** 10.3390/vaccines1020105

**Published:** 2013-04-10

**Authors:** Deanna Kruszon-Moran, R. Monina Klevens, Geraldine M. McQuillan

**Affiliations:** 1 Division of Health and Nutrition Examination Statistics, National Center for Health Statistics (NCHS), Centers for Disease Control and Prevention, 3311 Toledo Rd., Room 4308 Hyattsville, MD 20782, USA; 2 Division of Viral Hepatitis, National Center for HIV/AIDS, Viral Hepatitis, STD, and TB Prevention (NCHHSTP), Centers for Disease Control and Prevention,1600 Clifton Rd., MS G-37, Atlanta, GA 30333, USA; E-Mail: rmk2@cdc.gov; 3 Division of Health and Nutrition Examination Statistics, National Center for Health Statistics (NCHS), Centers for Disease Control and Prevention, 3311 Toledo Rd., Room 4204 Hyattsville, MD 20782, USA; E-Mail: gmm2@cdc.gov

**Keywords:** Hepatitis A virus, HAV, NHANES, vaccination, seroprevalence, birth cohort

## Abstract

To examine changes in seroprevalence of antibodies to hepatitis A virus (HAV) during a period in which universal vaccine recommendations for all U.S. children were implemented, results from serologic testing from the National Health and Nutrition Examination Survey (NHANES) from 2003–2010 were analyzed among 7,989 participants age 6–19 years, born in the U.S. in two birth cohorts (1986–1996 and 1997–2004). Overall prevalence increased over time from 24.4% in 2003–2006 to the highest ever reported (37.6%) in 2007–2010. Specifically, increases reached statistical significance in the birth cohort born in the years after implementation of vaccine recommendations (1997–2004), among those of race/ethnicity other than white, non-Hispanic, and among states where recommendations were implemented later. The greatest increase over time was among the subgroup of persons in states with early implementation who were of race/ethnicity other than white, non-Hispanic. Geographic region and birth cohort based on vaccine recommendations as well as race/ethnicity were the main predictors of seropositivity in 2007–2010. The increase in Hepatitis A seroprevalence occurred during a time of decreasing incidence and increasing vaccination, however race/ethnic disparities persist.

## 1. Introduction

Hepatitis A virus (HAV) is transmitted through the fecal-oral route and spread primarily through close personal contact with an HAV-infected person. Hepatitis A was once one of the most frequently reported notifiable diseases in the United States (U.S.) with a reported incidence of 10.7 cases per 100,000 population from 1990–1997, with incidence varying by age, gender, race/ethnicity and geographic region [1]. In 1995, the first Hepatitis A vaccines were licensed in the United States and by 1996 the Advisory Committee on Immunization Practices (ACIP) made recommendations for routine vaccination of children aged 2–18 years living in communities with the highest rates of infection and disease [2]. By 1999, epidemiologic evidence suggested that the strategy had a limited impact on national disease incidence [3]. Therefore in 1999, the ACIP recommended routine vaccination for children living in 11 mostly western states, with mean incidence rates that were at least twice the 1987–1997 national mean (*i.e*., ≥20 cases per 100,000 population). In addition, the ACIP recommended consideration of routine vaccination of children in an additional six states, where mean incidence rates were higher than the national average, but less than twice that value (*i.e*., 10–19 cases per 100,000 population [3]. By 2003, acute hepatitis A disease had declined overall by 76%, from a rate of 10.7 per 100,000 population during 1990–1997 to 2.6 per 100,000 population in 2003 [1]. In 2006, ACIP recommended integration of HAV vaccine into the routine childhood vaccination schedule, with HAV vaccine administered for all children at age 12 months [4]. By 2007, the rate of acute Hepatitis A again declined to 1.0 per 100,000 population [5] and by 2009 the rate was the lowest ever reported at 0.6 cases per 100,000 population [6]. The Healthy People 2020 goal is to reduce incident Hepatitis A cases to 0.3 cases per 100,000 population [7].

Population-based seroprevalence surveys play a critical role in supplementing data systems for disease incidence, vaccination coverage, and vaccine adverse events in the development of vaccination policy [8]. Before the availability of vaccine, seroprevalence of antibody to HAV (anti-HAV) in the population solely reflected prior infection [9]. Currently, seroprevalence can reflect immunity due to either previous infection or to vaccination. Earlier studies of data from the National Health and Nutrition Examination Surveys described HAV seroprevalence and predictors of seropositivity for the years prior to any vaccination (1988–1994) and prior to the 2006 ACIP recommendation of universal vaccination of all children (1999–2006) [9,10]. Studies of vaccination coverage show increased coverage since 2006, variability in coverage by race/ethnicity, higher coverage in the states where vaccine recommendations were initiated early (1999) but the greatest increase in coverage occurs among states in which vaccination recommendations were initiated later [11]. Our objective was to describe change in seropositivity to HAV in the U.S. among children and adolescents from the pre-vaccine era (2003–2006) to the post-vaccine era (2007–2010) for those age 6–19 years during a time of decreasing HAV incidence. We also evaluated sociodemographic factors associated with seroprevalence in the post vaccine era (2007–2010), and compared these findings with those of previous studies based on data before a vaccine became available.

## 2. Experimental Section

### 2.1. Data Source and Sample Design

Data for this study came from The National Health and Nutrition Examination Surveys (NHANES), a series of surveys conducted by the U.S. Centers for Disease Control and Prevention’s (CDC) National Center for Health Statistics that obtains nationally representative data on the health and nutritional status of the non-institutionalized, civilian population of the United States. NHANES uses a complex, stratified, multistage probability sample design and collects information using standardized household interviews, physical examinations conducted in mobile examination centers, and testing of biologic samples. The continuous NHANES began in 1999 and data files are released in two-year cycles. We analyzed data from eight years of the continuous NHANES, grouped into two four year cycles (2003–2006 and 2007–2010). For NHANES 2003–2006, non-Hispanic blacks, Mexican Americans, adolescents, and low income persons were sampled at higher frequencies than other persons to provide more precise estimates for these groups. Starting in 2007, adolescents are no longer oversampled and all Hispanic persons were targeted for oversampling rather than just Mexican American persons. More detailed information on survey design for NHANES surveys, including approval from the Ethics Review Board for data collection and analysis, is available from the survey documentation [12].

### 2.2. Laboratory Testing

Blood specimens from persons ≥6 years of age (for 2003–2006) and 6–19 years of age (2007–2010) were processed, stored, and shipped to CDC’s Division of Viral Hepatitis Laboratory. A qualitative determination of total anti-HAV in serum or plasma was measured using a solid-phase competitive enzyme immunoassay (HAVAB-EIA, Abbott Laboratories, Abbott Park, IL, USA). Serologic results for those age 6–19 years were used in this analysis.

### 2.3. Definitions the FIPR

An anti-HAV positive person was considered immune to HAV infection through either vaccination or natural infection. Race and ethnicity were categorized, based on a subjects’ self-reported information, as non-Hispanic white, non-Hispanic black, or Mexican American. Subjects that were not classified into one of these categories were classified as “other”. Country of birth was categorized as U.S. or non-U.S. birth. Poverty index was calculated by dividing family income by a poverty threshold specific for family size. The US Department of Health and Human Services’ poverty guidelines were used as the poverty measure to calculate the poverty index [13]. Education level was measured as last year of school completed using head of household education and grouped into three levels: less than a high school graduate, high school completed, and more than high school completed. Health insurance status was categorized as having any insurance or having none.

Analyses were restricted to U.S.-born persons to best reflect U.S.-acquired immunity. Based on restricted use NHANES geographic data, persons were grouped by state of residence into two groups, according to the 1999 ACIP hepatitis A vaccination recommendations and consistent with other analyses [3]: (1) the 17 “early vaccinating states”, where hepatitis A vaccination was recommended (AK, AZ, CA, ID, NM, NV, OK, OR, SD, UT, WA) or where vaccination was considered (AR, CO, MO, MT, TX, WY), and (2) the remaining 33 “later vaccinating states”, where no routine childhood vaccination was recommended in 1999 (AL, CT, DE, FL, GA, HI, KS, KY, IA, IL, IN, LA, MA, MD, ME, MI, MN, MS, NC, ND, NE, NH, NJ, NY, OH, PA, RI, SC, TN, VA, VT, WI, WV). Persons were also categorized into birth cohorts based on restricted data using actual date of birth and into two groups based on initiation of vaccine recommendations; those born between 1987–1996 (before vaccine recommendations) and those born between 1997–2004 (after vaccine recommendations). There were 936 sample persons age 16–19 born before 1987 in survey years 2003–2006 that were not included in either birth cohort and therefore were not included in this analysis. 

### 2.4. Statistical Analysis

Seroprevalence estimates were weighted to represent the total civilian, non-institutionalized U.S. household population and to account for oversampling and non-response to the household interview and physical examination [14]. Because we utilized a variable based on U.S. geography, we were unable to use the publically released masked stratum and primary sampling units (PSU’s) to designate the complex sample design in our analyses. The true stratum and PSU designations were used instead. Ninety-five percent confidence intervals (95% CI) were estimated by using the exact binary method [15]. Statistical comparisons between subgroups were evaluated using a *t-*statistic obtained from a linear contrast procedure in SUDAAN (release version 10.0, Research Triangle Institute, Research Triangle Park, NC, USA), a statistical package designed to analyze complex survey data. *P*-values of less than 0.05, with degrees of freedom equal to the minimum calculated for either subgroup in the comparison, were considered significant. No adjustments for multiple comparisons were made. Because prevalence was very low (less than 10%) in some smaller subgroups as well as very high (approaching 90%) in others, the stability of an estimate was based on both the percent positive and percent negative as well as the number of positive and negative individuals. An estimate was designated as unstable when the relative standard error of the estimate was greater than 30% (RSE = standard error of the percent/percent expressed as a percent) or when the number of negative or positive individuals was <10. Because of small numbers in each survey cycle for several race/ethnic subgroups when stratifying on geographic region, we collapsed race/ethnic categories to white, non-Hispanic and all other race/ethnic groups combined (all others).

A logistic modeling procedure in SUDAAN was used to evaluate interactions between change in seroprevalence by survey cycle and either race/ethnic group or birth cohort within each geographic region. Logistic modeling was also used to determine cofactors independently associated with anti-HAV seroprevalence for the data from 2007–2010. Model terms with a Satterthwaite-adjusted F statistic with a *p* < 0.05 were considered to be significant predictors of HAV seropositivity. 

## 3. Results

### 3.1. HAV Testing Response Rates

There were 4,955 persons aged 6–19 years born in the U.S. between 1987–2004, interviewed in NHANES 2003–2006, and 4,622 in NHANES 2007–2010. Ninety seven percent of those interviewed in both 2003–2006 (n = 4,788) and 2007–2010 (n = 4,488) were examined and 87% of those examined in 2003–2006 (n = 4,185) and 85% in 2007–2010 (n = 3,805) had blood drawn and were tested for Hepatitis A virus antibody. Response to HAV testing varied by many predictors of seropositivity including geographic region and race/ethnic group by ≤5%. Difference in response was greatest for birth cohort (89% for birth cohort 1987–1996 and 79% for birth cohort 1997–2004 in NHANES 2003–2006 and 89% and 80% respectively for birth cohorts 1987–1996 and 1997–2004 in NHANES 2007–2010, *p* < 0.001). All analyses were repeated with new weights adjusted for the differences in non-response by the three main predictors of seropositivity, geographic region, birth cohort and race/ethnic group. Estimates differed by less than 1% and there were no changes in any results. All results reported were calculated using original weights. 

### 3.2. Change in Seropositivity over Time (Survey Cycle) by Birth Cohort, Geographic Region, and Race/Ethnicity

Prevalence of anti-HAV among U.S. born individuals age 6–19 years increased over time by 13.1 percentage points from 24.4% (95% CI 16.6–33.9%) in NHANES 2003–2006 to 37.6% (95% CI 32.6–42.7%) in NHANES 2007–2010 (*p* < 0.05) (Table 1). Prevalence of antibody increased over time in the region with later vaccine recommendations (11.5 percentage points, *p* < 0.01). A similar effect was found in the region with early recommendations (18.5 percentage points) but it did not reach statistical significance. Overall seropositivity was significantly higher in the region with earlier recommendations as compared to the region with later recommendations in both four year survey cycles (*p* < 0.001 for both). 

Prevalence increased significantly over time in the post vaccine birth cohort (1997–2004) (18.9 percentage points *p* < 0.01) (Table 1). There was not a significant increase in prevalence over time among persons in the pre-vaccine birth cohort (1987–1996). Prevalence was significantly higher in the post vaccine birth cohort as compared to the pre-vaccine birth cohort but only in the later survey cycle (*p* < 0.001). 

Prevalence increased significantly over time among non-Hispanic blacks (19.1 percentage points, *p* < 0.01) and among Mexican Americans (23.3 percentage points, *p* < 0.001) as well as among all persons combined who were not non-Hispanic white (24.0 percentage points, *p* < 0.001). There was no increase in prevalence among white, non-Hispanic persons (Table 1). Seroprevalence was lower among white, non-Hispanic persons compared to all others combined and compared to Mexican Americans in both survey cycles (Table 1). Seroprevalence was also lower among white, non-Hispanic persons as compared to black, non-Hispanic persons but only in the 2007–2010 survey cycle (*p* < 0.01). Black, non-Hispanic persons had lower seroprevalence compared to Mexican Americans in both survey cycles (*p* < 0.001 for both).

Because change in seropositivity over time and initiation of vaccine recommendation policy were both strongly associated with geographic region, additional analyses were conducted stratified on region. Due to small numbers and unstable estimates with the multi-level stratification needed to test for these interactions, differences in race/ethnicity were limited to the two category variable for these analyses—white, non-Hispanics and all others combined. Significance tests for change over time and possible interactions with race/ethnicity or year of birth cohort were conducted using logistic regression stratifying on geographic region and adjusting for the other cofactor. 

**Figure 1:**
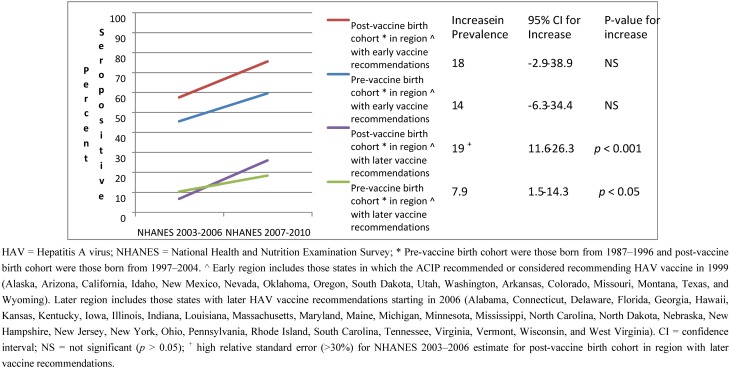
HAV seropositivity over time among U.S. born children and adolescents 6–19 years old stratified by birth cohort and region of the U.S.: NHANES 2003–2006 and 2007–2010.

The increase in seropositivity over time, although it did not reach statistical significance, was similar in both birth cohorts in the geographic region with early recommendations (*p* > 0.05 for both, *p* > 0.05 for interaction term, Figure 1). This was not true in the region with later recommendations. In the later region, the increase over time was greater in the post-vaccine birth cohort (*p* < 0.001) as compared to the pre-vaccine cohort (*p* < 0.05) (*p* < 0.001 for the interaction term). Note the relative standard error for the estimate for the first survey cycle for those in the post vaccine birth cohort in this region was high (>30%) making the estimate unstable; therefore, results should be interpreted with caution. 

**Figure 2:**
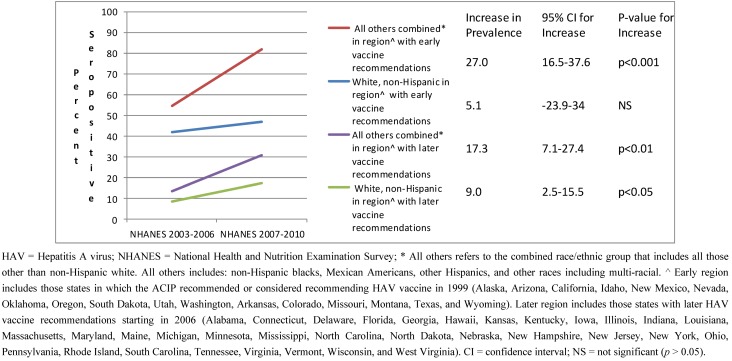
HAV seropositivity over time among U.S. born children and adolescents 6–19 years old stratified by race/ethnic group and region of the U.S.: NHANES 2003–2006 and 2007–2010.

The increase in seropositivity was similar between white, non-Hispanics (*p* < 0.05) and all others combined (*p* < 0.01) in the geographic region with later vaccine recommendations (Figure 2). There was no significant interaction between change in seropositivity (survey cycle) and race/ethnic group in this region (*p* > 0.10 for interaction term). In contrast, in the region with early vaccine recommendations, the increase in seropositivity over time was much greater among the all others combined race/ethnic group (*p* < 0.001) as compared to the white, non-Hispanic race/ethnic group (*p* > 0.05) (*p* < 0.05 for the interaction term). 

### 3.3. Predictors of Seropositivity Using Logistic Models for 2007–2010

To determine significant socio-demographic predictors of current HAV antibody seropositivity in the U.S. for the most recent point in time, data from the 2007–2010 survey cycle was analyzed using logistic regression modeling. Possible predictors evaluated were determined from previous analyses published using 1999–2006 data [10]. Significant predictors were defined as those with a *p* < 0.05 in the full model (Table 2). As expected geographic region based on early vaccine recommendations, year of birth cohort and race/ethnicity were significant predictors of seropositivity. Additional predictors of greater seropositivity included female gender.

Because vaccine recommendations were initiated at different times by region of the U.S. and changes in seropositivity over time by race and birth cohort also varied by region, models were run stratified by this variable. Higher seropositivity in 2007–2010 among those in the all others combined race/ethnic group as compared to white, non-Hispanic sample persons was much greater in the geographic region with early vaccine recommendations (odds ratio (OR) 5.1 (95% CI 2.2–11.6) as compared to the region with later recommendations (OR 2.2 (95% CI 1.5–3.2). Other than race/ethnicity, year of birth cohort was the only other significant predictor in the region with early vaccine recommendations. There were no other significant predictors of seropositivity for this region. Possible interactions of each cofactor with year of birth cohort were also examined but estimates were too unstable in many subgroups to stratify on year of birth cohort. 

**Table 1:** Prevalence of HAV antibody among U.S. born 6–19 year old children and adolescents: NHANES 2003–2006 and 2007–2010.

		NHANES 2003–2006				NHANES 2007–2010				
	n	Percent	Lower	Upper	n	Percent	Lower	Upper	Increase	*p*-value
		Prevalence	95% CI	95% CI		Prevalence	95% CI	95% CI	Over time	
All	4,185	24.4	16.6	33.9	3,804	37.6	32.6	42.7	13.1	*p* < 0.05
Region of the U.S. *										
Early region (ref)	1,759	47.4	34.8	60.3	1,600	66.0	52.2	78.0	18.5	NS
Later region	2,426	10.1 ^a^	7.3	13.5	2,204	21.6^a^	16.5	27.4	11.5	*p* < 0.01
Birth Cohort ^										
Pre-vaccine (ref)	3,682	24.4	16.4	34.1	2,023	33.8	28.8	39.0	9.3	NS
Post vaccine	503	24.5	16.1	34.8	1,781	43.4 ^a^	38.0	48.9	18.9	*p* < 0.01
Race/ethnicity										
White, non-Hispanic (ref)	1,162	20.1	10.4	33.2	1,258	25.5	20.9	30.7	5.4	NS
Black, non-Hispanic	1,508	20.0	13.9	27.3	939	39.1 ^b^	30.8	47.9	19.1	*p* < 0.01
Mexican American	1,190	53.2 ^a^	47.2	59.1	971	76.5 ^a^	67.6	83.9	23.3	*p* < 0.001
White, non-Hispanic (ref)	1,162	20.1	10.4	33.2	1,258	25.5	20.9	30.7	5.4	NS
All others ^+^	3,023	32.0 ^c^	26.7	37.6	2,546	56.0 ^a^	49.0	62.8	24.0	*p* < 0.001

HAV = Hepatitis A virus; NHANES = National Health and Nutrition Examination Survey; CI = confidence interval; * Early region includes those states in which the ACIP recommended or considered recommending HAV vaccine in 1999 (Alaska, Arizona, California, Idaho, New Mexico, Nevada, Oklahoma, Oregon, South Dakota, Utah, Washington, Arkansas, Colorado, Missouri, Montana, Texas, and Wyoming). Later region include those states with later HAV vaccine recommendations starting in 2006; (Alabama, Connecticut, Delaware, Florida, Georgia, Hawaii, Kansas, Kentucky, Iowa, Illinois, Indiana, Louisiana, Massachusetts, Maryland, Maine, Michigan, Minnesota, Mississippi, North Carolina, North Dakota, Nebraska, New Hampshire, New Jersey, New York, Ohio, Pennsylvania, Rhode Island, South Carolina, Tennessee, Virginia, Vermont, Wisconsin, and West Virginia); ref = reference group; NS = not statistically significant; ^a^ = *p* < 0.001, ^b^ = *p* < 0.01, ^c^ = *p* < 0.05 from t-test comparing subgroup to reference group within each survey cycle. ^ Pre-vaccine birth cohort were those born from 1987–1996 and post vaccine birth cohort were those born from 1997–2004. ^+^ All others refers to the combined race/ethnic group that includes all those other than non-Hispanic white. All others includes non-Hispanic blacks, Mexican Americans, other Hispanics, and other races including multi-racial.

**Table 2:** Predictors of HAV seropositivity from logistic regression models among U.S. born children and adolescents 6–19 years of age by region based on HAV vaccine recommendations and birth cohort: NHANES 2007–2010.

					Pre-vaccine	Post-vaccine
					Birth cohort in	Birth cohort in
		All	Region with Early Vaccine	Region with Later Vaccine	Region with Later Vaccine	Region with Later Vaccine
			Recommendations ^a^	Recommendations ^b^	Recommendations	Recommendations
Characteristics		OR (95% CI)	OR (95% CI)	OR (95% CI)	OR (95% CI)	OR (95% CI)
Region of the U.S.						
	Early ^a^	6.7 (3.5–13.0) ^c^	NA	NA	NA	NA
	Later ^b^	ref				
Birth Cohort *						
	Pre-vaccine	ref	ref	ref	NA	NA
	Post-vaccine	1.6 (1.4–2.0) ^c^	1.9 (1.2–2.8)^e^	1.5 (1.3–1.7) ^c^		
Race/ethnicity						
	Non-Hispanic white	ref	ref	ref	ref	ref
	All others ^	3.0 (2.0–4.6) ^c^	5.1 (2.2–11.6)^c^	2.2 (1.5–3.2) ^c,f^	1.5 (1.0–2.4)	3.2 (2.2–4.8) ^c^
Gender						
	Male	ref	ref	ref	ref	ref
	Female	1.2 (1.0–1.5) ^d^	1.0 (0.7–1.4)	1.3 (1.1–1.6)^e^	1.3 (0.9–1.9)	1.4 (1.1–1.8) ^d^
Poverty Index						
	<1.0	1.0 (0.8–1.2)	1.0 (0.7–1.5)	1.0 (0.7–1.2)	1.0 (0.7–1.4)	0.9 (0.6–1.3)
	≥1.0	ref	ref	ref	ref	ref
Education of head of household						
	<High school	1.1 (0.8–1.6)	1.0 (0.6–1.8)	1.1 (0.8–1.6)	1.0 (0.6–1.6)	1.3 (0.8–2.2)
	High school graduate	0.8 (0.5–1.1)	0.9 (0.5–1.6)	0.7 (0.5–1.1)^f^	0.5 (0.3–0.9) ^d^	1.0 (0.6–1.8)
	>High school	ref	ref	ref	ref	ref
Household size						
	≤4 persons	ref	ref	ref	ref	ref
	5 or more persons	1.3 (1.0–1.6)	1.3 (0.9–1.9)	1.2 (0.8–1.8)	1.2 (0.7–2.2)	1.1 (0.8–1.6)
Health Insurance						
	Any	1.7 (1.0–2.8)	1.6 (0.7–3.8)	2.0 (1.2–3.3) ^d^	1.9 (1.0–3.7) ^d,r^	1.9 (0.7–4.8)
	None	ref	ref	ref	ref	ref

HAV = Hepatitis A virus; NHANES = National Health and Nutrition Examination Survey; OR = odds ratio; CI = confidence interval. ^a^ Early region includes those states in which the ACIP recommended or considered recommending HAV vaccine in 1999 (Alaska, Arizona, California, Idaho, New Mexico, Nevada, Oklahoma, Oregon, South Dakota, Utah, Washington, Arkansas, Colorado, Missouri, Montana, Texas, and Wyoming). ^b^ Later region includes those states with later HAV vaccine recommendations starting in 2006 (Alabama, Connecticut, Delaware, Florida, Georgia, Hawaii, Kansas, Kentucky, Iowa, Illinois, Indiana, Louisiana, Massachusetts, Maryland, Maine, Michigan, Minnesota, Mississippi, North Carolina, North Dakota, Nebraska, New Hampshire, New Jersey, New York, Ohio, Pennsylvania, Rhode Island, South Carolina, Tennessee, Virginia, Vermont, Wisconsin, and West Virginia). NA = not applicable; ref = reference group. * Pre-vaccine birth cohort were those born from 1987–1996 and post-vaccine birth cohort were those born from 1997–2004. ^ All others refers to the combined race/ethnic group that includes all those other than non-Hispanic white. All others includes: non-Hispanic blacks, Mexican Americans, other Hispanics, and other races including multi-racial. ^c^ *p* < 0.001 for comparison of subgroup and reference group in model; ^d^ *p* < 0.05 for comparison of subgroup and reference group in model; ^e^ *p* < 0.01 for comparison of subgroup and reference group in model; ^f^ *p* < 0.05 for interaction of cofactor and year of birth cohort within geographic region; ^r^ = RSE > 30% in univariate analysis—estimates may be unstable.

In the region with later vaccine recommendations, besides race/ethnicity, post vaccine birth cohort, female gender and having health insurance were associated with greater seropositivity to HAV. In addition, a significant interaction with birth cohort and both race/ethnicity and head of household education was found. Logistic models for this region stratified by birth cohort demonstrated larger race/ethnic differences (higher seropositivity among all others ) in the post-vaccine birth cohort (OR 3.2 (95% CI 2.2–4.8), *p* < 0.001) as compared to the pre-vaccine birth cohort (OR 1.5 (95% CI 1.0–2.4), *p* > 0.05). There was no difference between those whose head of household had less than a high school education as compared to those with greater than a high school education in either cohort. Individuals whose head of household educational level was equal to high school had lower seropositivity than those with greater than a high school education but this was only true in the pre-vaccine cohort (*p* < 0.05).

## 4. Discussion

We report here the highest ever prevalence of immunity to hepatitis A among U.S. children (37.6%) through 2010; prevalence was especially high (66%) among children residing in areas with an early recommendation (1999) or consideration of vaccination. This prevalence is almost five times higher than the 8% level found in children during the 1988–1994 NHANES [9], *i.e*., before the vaccine became available. Given the steady decline in disease incidence [16] our findings of an increase in seroprevalence are consistent with the observed increase in vaccination coverage [17]. 

In a previous NHANES study period, pre-dating vaccination, children who lived in poverty, in households of less educated adults, or in crowded homes, were more frequently immune because of infection [9]. In this study of U.S. born children in NHANES 2007–2010, we document that socio-economic factors were not associated with immunity. This might be related to the Vaccines for Children Program (VFC), which has been providing vaccines at no cost to children in families unable to pay, since 1994 [18]. 

The increase in seroprevalence between 2003–2006 and 2007–2010 was similar in both the region with early vaccine recommendations as compared to the region with later vaccine recommendations although it only reached statistical significance in the latter region. Although not statistically significant, the change in seroprevalence in both birth cohorts in the region with early recommendations were similar in magnitude to the significant increase found in the post vaccine birth cohort in the region with later vaccine recommendations. The lack of statistical significance in the early region was partly due to the fact that there were fewer sample persons and fewer stratum and PSU’s in this region thereby resulting in larger standard errors for these estimates and less power to detect statistical significance. The increase in seroprevalence was similar between the two birth cohorts in the region with early recommendations. In contrast, in the region with later recommendations, the increase in seroprevalence was greater in the post-vaccine birth cohort. There appeared to be no difference in prevalence of antibody by birth cohort in this region in 2003–2006 but because of the greater increase in seroprevalence in the post-vaccine cohort, differences between birth cohorts were seen in 2007–2010. The smaller change in seroprevalence in the cohort born before implementation in the region with later recommendations may be due to a variety of factors including: older age of this cohort at the time vaccine recommendations were implemented and the fact that initial recommendations were risk-based and this group may not have been perceived to be at risk.

We did observe one disparity: children residing in areas with early recommendations in the all other combined race/ethnic group had a significantly higher prevalence of immunity (82% by 2007–2010) and a significantly greater increase in seroprevalence compared to white, non-Hispanic children. In 2007–2010, controlling for other variables, children in the all others combined race/ethnic group in states with an early vaccination recommendation were 5 times more likely to be protected from hepatitis A than white, non-Hispanic children. In regions with later recommendations, these children were 2 times more likely to be immune to hepatitis A. These findings are consistent with a survey of vaccination coverage among teenagers in 2009 [19] that found that coverage was low overall (two doses 30%) and that white, non-Hispanic teens had the lowest coverage of all racial/ethnic groups (66% in states with early recommendations and 25% in states with no recommendation in 1999). No such disparity has been observed among infants. Vaccination coverage with 2 doses was not significantly different between white and black, non-Hispanic infants; however, coverage among Hispanic and Asian infants was significantly higher than among white, non-Hispanic children [17]. Self-reported vaccination with hepatitis A vaccine was similar among white, black, and Hispanic adults aged 19–49 years in the 2010 National Health Interview Survey [20]. It is possible that the perception of risk for hepatitis A among white children is lower than among children in other race/ethnic groups. 

The United States is not alone in adopting hepatitis A vaccination with success. Israel was the first country to initiate routine vaccination with a two-dose regimen of hepatitis A vaccine in 1999. There, the incidence of hepatitis A dropped over 95% in children and adolescents by 2007 [21]. In Argentina, the introduction of a single dose regimen for children 12 months of age was followed by an 83% reduction in the average incidence rate by 2007 [22], In Puglia (Southern Italy), vaccination of toddlers and preadolescents was initiated in 1998 after an HAV epidemic in the years 1996–1997. Incidence of HAV infection declined by 95% by 2009 due to both vaccination and reduced circulation although presence of antibody to HAV remained low in some age groups [23].

A strength of this study was that there were sufficient data to allow a more in-depth analysis of immunity within geographic areas with and without early vaccination recommendations (*i.e*., to vaccinate in 1999) as well as by the birth cohorts effected by these recommendations. A limitation of all NHANES data are that findings are generalizable to the U.S. non-institutionalized civilian population of the United States, and do not represent persons residing in institutions (e.g., illicit drug users in prisons). A limitation of the assay used to detect antibodies to HAV is that they are unable to distinguish immunity related to infection or to vaccination.

## 5. Conclusions

In summary, U.S. children aged 6–19 years through 2010 had the highest ever prevalence of immunity to hepatitis A (37.6%) and immunity reached 66% among children in the region with early recommendations. Prevalence increased over time and this increase was greatest among those of the all other race/ethnic group other than non-Hispanic white. Continued monitoring of immunity, vaccination coverage, and incidence of disease will help guide next steps in the U.S. hepatitis A vaccination policy.

The findings and conclusions in this paper are those of the author(s) and do not necessarily represent the official position of the National Center for Health Statistics, Centers for Disease Control and Prevention. 
